# Gene action and heterosis in F_1_ clonal progenies of cassava for β-Carotene and farmers’ preferred traits

**DOI:** 10.1016/j.heliyon.2019.e01807

**Published:** 2019-06-17

**Authors:** Nduwumuremyi Athanase, Melis Rob

**Affiliations:** a Rwanda Agriculture and Animal Resources Development Board (RAB), Kigali, P.O. Box 5016, Rwanda; b University of KwaZulu-Natal, African Centre for Crop Improvement (ACCI), Scottville, Private Box X01, 3029, South Africa

**Keywords:** Genetics

## Abstract

Gene action and heterosis provides information to assist breeder for selecting and generating improved plant recombinants. This study aimed at determining the gene action of selected cassava traits. The F_1_ clones exhibited considerable phenotypic variability between families and offsprings. The best F_1_ progenies had a higher amount of β-carotene (β-C) of 6.12 mg 100 g^−1^ against 1.32 mg 100 g^−1^ of the best parent. This superiority could be attributed to the over-dominance from the recombination of additive gene action and epistasis. The general combining ability (GCA) of parents and specific combining ability (SCA) of combinations were significant for different traits, and indicating the role of additive and non-additive gene action in controlling such traits. The significant GCA for β-C and postharvest physiological deterioration (PPD) indicates the role of additive gene action. The significant SCA for cassava mosaic disease (CMD) and cassava brown streak disease (CBSD) showed a predominance of non-additive gene action. The F_1_ progenies from the family Mavoka x Garukunsubire expressed the highest positive heterosis for CMD, dry matter and β-C. The high positive heterosis for β-C and DMC could be linked to transgressive segregation, because one of the parents was poor combiner.

## 1 Introduction

Cassava is a cash crop and generates income for smallholder farmers in many countries of tropical and subtropical Africa, Asia, and Latin America. In Africa, its annual per capita consumption is around 80 kg per person (IITA, 2016). In sub-Saharan Africa (SSA), cassava is mainly a subsistence crop for small-scale farmers. In Rwanda, the preferred cassava traits are a sweet taste, a high yield, good quality ugali (viscosity and colour), resistance to pests and diseases, early bulking, multipurpose, good colour of flesh and flour, delayed post-harvest physiological deterioration (PPD), high dry matter content, good odour/smell, long storage ability in the field, many cuttings produced and good cooking ability (Nduwumuremyi et al., 2016a, Nduwumuremyi et al., 2016b).

The viral diseases and postharvest losses are the most serious challenges for cassava production in developing countries. Cassava brown streak disease (CBSD) and cassava mosaic disease (CMD) affect the cassava yield and storage root quality in most parts of East Africa (Ephraim et al., 2015; Rwegasira and Rey, 2012). In addition, PPD causes significant postharvest losses, as the storage root perish rapidly (Kiaya, 2014). Cassava breeding is the most sustainable strategy to generate new high yielding recombinants that are resistant to diseases, with delayed physiological deterioration. During the breeding process, information generated on the combining ability and heterosis assists in the development of new improved recombinants (Mendes et al., 2015; Zhao et al., 2016). At present, there is limited genetic information on the combining ability and heterosis for yield, postharvest and quality, disease traits, and other important cassava traits in Rwanda.

To generate new recombinants, the half-diallel mating design is commonly used by cassava breeders (Nduwumuremyi et al., 2013; Tumuhimbise, 2013). The diallel analysis provides information on the general combing ability (GCA), the specific combining ability (SCA) and heterosis (Glover et al., 2005). The combining ability indicates the capacity to transmit characteristics from parents to offspring (Upadhyay and Jaiswal, 2015). A knowledge of the combining ability helps to determine gene action and to identify the best genotypes (parents) for hybridization, as well as the identification or selection of the best combinations (crosses) for population improvement. This information is very important for designing suitable breeding strategies for cassava improvement. Therefore, this study aimed at determining the gene action of F_1_ cassava progenies for cassava β-carotene, delayed postharvest physiological deterioration, and other farmers preferred traits.

## 2 Materials and methods

### 2.1 Description of experimental locations

The experiment was conducted in Rwanda at two locations, namely, the Karama and Muhanga research centres. Karama is located at 2^o^16′ 0.927″S, 30^o^15′ 20.693″E, with an altitude of 1332 m above sea level m (asl), while Muhanga is located at 2^o^04′9.842″S, 29^o^43′ 9.842″E, with an altitude of 1879 masl. The two locations experience bimodal rainfall with different amounts of rain and temperatures. The Muhanga location is cooler and receives a higher amount of rain (1320 mm per year) than the Karama location (895 mm per year).

### 2.2 Parent selection, hybridization, experimental design and management

Six genotypes (Table 1) were selected from research institutes, farmer's cooperatives and private farms. The selection of genotypes was done in a participatory manner through consultative discussion between local scientists and farmers. The main traits for selection were high yield, cassava brown streak disease (CBSD) resistance and pulp colour. The parents were planted in crossing block paired rows at the Karama research station, located at 2^o^15′54.126″S, 30^o^15′22.4619″E, with an altitude of 1338 m asl. The 6 × 6 half-diallel was produced to generate fifteen families. Hand pollination was performed following the procedure proposed by Kawano (1980). The 450 clones were selected from the fifteen families seedling plants with an equal number (30) of clones per family. The clonal trial planted in October 2015 in randomised complete block design (RCBD) with three replications. The population density was 10000 plants ha^−1^ (1 × 1 m spacing). The weeding was conducted regularly and ridging was performed once, three months after planting, while no fertilizers, pesticides and water irrigation were applied.

**Table 1:** Descriptions of parental germplasm.

Code of genotypes	Name of genotypes	Type	Yield	CMD	CBSD	Pulp colour
G1	Mavoka	Improved	High	Resistant	Susceptible	Yellow
G2	Garukansubire	Improved	High	Resistant	Susceptible	Yellow
G3	Gahene	Landrace	High	Susceptible	Susceptible	White
G4	Mushedile	Landrace	High	Susceptible	Tolerant	White
G5	Ndamirabana	Improved	High	Resistant	Susceptible	White
G6	Gitamisi	Landrace	High	Susceptible	Tolerant	White

CMD: cassava mosaic disease, CBSD: cassava brown streak disease.

### 2.3 Data collection

At 3 and 6 months after planting (MAP), and at harvest the data were collected on each individual plant for cassava mosaic disease (CMD), cassava brown streak disease (CBSD) severity, and cassava brown streak disease root necrosis (CBSD-RN), scored on a scale of 1–5, where: 1 = no symptoms; and 5 = very severe symptoms (Hillocks et al., 1996). The fresh root storage yield (FSRY) (t ha^−1^) was estimated from storage root mass per plant. To estimate FRSY, the storage root mass (SRM) was used following the formula:

$$FRSY\;\left(kg\;ha^{-1}\right)=SRM\;\left(kg\;plant^{-1}\right)\times \;\frac{10000\;}{1000}$$

The harvest index (HI) was determined by expressing fresh storage root mass (kg plant^−1^) as proportion of total biomass (kg plant^−1^) (Fukuda et al., 2010). The DMC was determined by using the specific gravity method (Chávez et al., 2005; Fukuda et al., 2010; Kawano et al., 1987), as per the following formula:

$$\;\text{DMC}\;\left(\%\right)=\left(\frac{WA}{WA-WW}\times 158.3\right)-142$$

Where WA and WW are weight measured in air and water, respectively.

The β-carotene (β-C) was estimated using the colour chart that was used in estimating β-C in sweet potatoes, as described by Burgos et al. (2009). The PPD was determined using the method developed by CIAT (Morante et al., 2010; Zidenga et al., 2012). The proximal and distal ends of cassava storage roots were removed immediately after harvesting. Proximal ends were exposed to the air and distal ends of the storage root were covered, using food plastic wrappers. The room temperature ranged from 22-28 °C and the relative humidity was between 70-80%. The assessment was conducted seven days after harvest, using the score 1–10 to represent the discoloration, where score 1 = 10%, 2 = 20%, 10 = 100% (Chávez et al., 2005; Wheatley et al., 1985). The two storage roots per genotype were cut into ten transversal slices, each 2 cm thick, and the mean score was performed on 20 slices from the two selected storage roots.

### 2.4 Data analysis

Data collected from individual plant which constitute a family, were averaged for statistical analysis using SAS studio (University edition). The GCA and SCA effects were estimated according to Griffing's (1956b) Model 1, Method 4 using the DIALLEL-SAS05 program developed by Zhang et al. (2005). The significance was expressed at p < 0.05, 0.01 and 0.001. The GCA and SCA effects for each trait were determined from the percentage of families' sum of squares (SS) due to GCA and SCA (Tumuhimbise et al., 2014). The relative importance of the GCA and SCA effects for each trait was determined from the percentage of the families' sum of squares (SS) (Tumuhimbise, 2013; Were et al., 2012). The mid-parent (MP) and best parent (BP) heterosis was analysed, using the formula $\text{MPH}\left(\%\right)=\frac{\text{(}\;\text{F}1-\text{MP)}}{\text{MP}}\times \;100$, and $\text{BPH}\;\left(\text{\%}\right)\;=\;\frac{\text{(}\;\text{F}1-\text{BP)}}{\text{BP}}\times \;100$. The selection of the best clones for advancement was done by using the selection index (SI) proposed by Ceballos et al. (2004), with some modifications. SI=(FRSY*5)+(β−Carotene*4)−(PPD*3)−(CBSD−RN*2), and the variables were standardized, using the following formula:Xi=(Xi−μ)/St.Dev.

## 3 Results

### 3.1 Descriptive statistics of ten selected traits of F_1_ cassava clones

The selected cassava traits evaluated in this study, showed a considerable variation among the F_1_ clones of fifteen families generated through 6 × 6 half-diallel mating design. The FSRY, β-C and TBM were highly skewed, while the CMD-S and HI were moderately skewed, explaining the genetic diversity among the generated F_1_ clones. The FSRY ranged from 2.0 to 44.2 t ha^−1^, with an average of 8.7 t ha^−1^, while DMC ranged from 26.1 to 42.1%, with an average of 34.0%. The β-C ranged from 0.001 to 1.88 mg 100g^−1^, with an average of 0.34 mg 100g^−1^, while the PPD evaluated showed a deterioration of 10–60.5% after one week (Table 2).

**Table 2:** Summary statistics of 10 traits measured in clonal F_1_ of 15 cassava families evaluated at two sites.

Traits	Mean	SE	SD	Kurtosis	Skewness	Minimum	Maximum
CMD-S	1.49	0.05	0.44	1.21	0.98	1.00	3.10
CBSD-L	2.01	0.03	0.31	-0.30	-0.27	1.20	2.67
CBSD-S	2.18	0.05	0.46	0.04	-0.25	1.00	3.29
CBSD-RN	2.14	0.09	0.87	-1.51	0.14	1.00	3.50
FSRY	8.70	0.67	6.32	10.17	2.47	1.98	44.20
TB	2.66	0.11	1.02	1.15	1.09	1.07	6.45
HI	0.30	0.01	0.10	1.99	0.78	0.10	0.69
DMC	33.97	0.35	3.36	-0.09	-0.49	26.10	42.10
β-C	0.32	0.05	0.45	2.83	1.79	0.001	1.88
PPD	33.85	1.51	14.37	-0.91	0.26	10.00	60.55

SE: standard Error, SD: standard deviation, CMD-S: cassava mosaic disease severity, CBSD-L: cassava brown streak disease on leaves, CBSD-S: cassava brown streak disease on stem, CBSD-RN: cassava brown streak disease root necrosis, FSRY: fresh storage root yield, TB: total biomass, HI: harvest index, DMC: dry matter content, β-C: β-Carotene, PPD: postharvest physiological deterioration.

### 3.2 Diallel analysis for β-carotene, delayed postharvest physiological deterioration and farmers’ preferred traits of cassava

The environment (E) significantly (*p* < 0.001) influenced the expression of all traits, except CMD, DMC and PPD. The families exhibited significant differences for CBSD-S, FSRY and HI at p < 0.05 and at p < 0.001 for the remaining traits. The effects due to families were further partitioned into two components, namely, the effects of GCA and SCA. The GCA was significant at p < 0.05 for CMD, DMC, and significant at p < 0.001 for β-C and PPD. The SCA was significant for β-C and PPD at p < 0.001, CMD and DMC at p < 0.01, and TB and CBSD-L at p < 0.05.

The relative importance of additive and non-additive gene actions for the expression of the studied traits were partitioned into GCA and SCA effects, expressed as a percentage of the sum of squares. The variability of the trait expression among families indicated that the pulp traits (CBSD-RN, β-C and PPD) were highly influenced (over 50% of variability) by the GCA effects, while CMD, CBSD-L, CBSD-S, TB, FSRY, HI and DMC, leaves and yield traits were considerably influenced (over 50% of variability) by the SCA effects (Table 3).

**Table 3:** Combined analysis of variance for important traits of 15 families of cassava clones generated from 6 × 6 half-diallel.

Source of variation	Mean squares					
	DF	CMD	CBSD-L	CBSD-S	CBSDN	TB
Environments (E)	1	0.06	1.20***	5.34***	56.64***	25.26***
Families	14	0.42***	0.16***	0.19*	0.21***	1.73***
E x Families	14	0.36***	0.18***	0.34***	0.36***	1.77***
GCA	5	0.37*	0.09	0.24	0.32	0.68
SCA	9	0.44**	0.18*	0.16	0.14	2.31*
Error	74	0.10	0.04	0.09	0.05	0.32
CV (%)		22.03	10.01	13.89	10.06	21.3
% SS due to GCA		31.6	22.6	46.3	55.8	14.1
% SS due to SCA		68.4	77.4	53.7	44.1	85.9
Source of variation	Mean squares					
	DF	FSRY	HI	DMC	β-C	PPD
Environments (E)	1	705.31***	0.21***	0.64	0.35***	57.36
Families	14	51.27*	0.01*	26.08***	1.00***	912.69***
E. Families	14	58.28**	0.00	15.45*	0.13***	35.50
GCA	5	34.16	0.01	23.13*	2.31***	1564.96***
SCA	9	60.78	0.00	27.71**	0.27***	550.32***
Error	74	21.24	0.00	7.38	0.02	84.46
CV (%)		52.9	24.12	7.99	46.72	27.15
% SS due to GCA		23.8	42.1	31.7	89.5	61.2
% SS due to SCA		76.2	57.9	68.3	10.5	38.5

GCA: general combining ability, SCA: specific combining ability, CV: coefficient of variation, %SS: percentage sum of squares, CMD-S: cassava mosaic disease severity, CBSD-L: cassava brown streak disease on leaves, CBSD-S: cassava brown streak disease on stem, CBSD-RN: cassava brown streak disease root necrosis, FSRY: fresh storage root yield, TB: total biomass, HI: harvest index, DMC: dry matter content, β-C: β-Carotene, PPD: postharvest physiological deterioration.

### 3.3 General combining ability for β-carotene, delayed postharvest physiological deterioration and other farmers’ preferred traits of cassava

The GCA effects of the parents were determined for the measured traits. Mavoka had a significant (p < 0.001) positive GCA for β-C, an undesirable significant (p < 0.01) negative GCA for DMC, and desirable significant (p < 0.001) negative GCA for PPD. Garukunsubire also showed significant (p < 0.001) positive GCA for β-C and significant (p < 0.001) negative GCA for PPD. The GCA for Mavoka and Garukunsubire indicates the ability of both parents to improve the level of β-C and delayed PPD. Gahene, Ndamirabana and Gitamisi showed significant (p < 0.001) negative GCA for β-C and positive GCA for PPD, while Mushedile had significant negative GCA for β-C (Table 4). In addition, Mavoka had the highest mean for β-C, followed by Garukunsubire.

**Table 4:** Means and general combining ability effects for important traits of six parents of cassava clones of 6 × 6 half-diallel families.

Parents	CMD		CBSD-L		CBSD-S		CBSD-RN		FSRY (t ha^−1^)	
	$\bar{\text{X}}$	GCA	$\bar{\text{X}}$	GCA	$\bar{\text{X}}$	GCA	$\bar{\text{X}}$	GCA	$\bar{\text{X}}$	GCA
Mavoka	1	-0.10	4	0.05	3	0.06	3	0.16	17.3	1.41
Garukunsubire	1	0.02	4.5	0.00	4	-0.07	4.5	-0.16	12.6	0.72
Gahene	4.5	0.02	4	-0.02	4	0.00	4	-0.08	2.4	-0.85
Mushedile	3.5	0.22**	2.5	-0.10*	1	-0.14	1	-0.00	26.3	-1.81
Ndamirabana	1.5	-0.05	2	0.01	3	0.00	2	0.08	19.3	-0.20
Gitamisi	3	-0.11	3	0.07	2	0.14	3	0.00	9.13	0.73
Means	2.4		3.3		2.8		2.9		14.5	
SE	0.3		0.2		0.2		0.3		1.9	

Parents	TB		HI		DMC (%)		βC (mg 100g-1)		PPD (%)	
	$\bar{\text{X}}$	GCA	$\bar{\text{X}}$	GCA	$\bar{\text{X}}$	GCA	$\bar{\text{X}}$	GCA	$\bar{\text{X}}$	GCA
Mavoka	5.5	0.15	0.31	0.00	26.0	-1.72**	1.32	12.27***	10.	-11.72***
Garukunsubire	5.5	0.15	0.23	0.01	29.4	0.23	0.03	6.59***	55.	-7.30***
Gahene	0.9	-0.24	0.27	0.00	33.1	0.68	0	-7.01***	50.	5.24**
Mushedile	5.6	-0.15	0.47	-0.04*	32.5	1.12*	0	-4.01***	55.	9.98***
Ndamirabana	5.2	-0.00	0.37	-0.00	31.3	-0.25	0	-4.01***	40.	1.27
Gitamisi	4.5	0.09	0.2	0.01	33.5	-0.06	0	-3.82***	50.	2.52
Means	**4.5**		***0.31***		***30.9***		***0.25***		***43.3***	
SE	**0.4**		***0.02***		***0.6***		***0.12***		***3.8***	

SE: standard error, $\bar{\text{X}}$: means**,**GCA: general combining ability, CMD-S: cassava mosaic disease severity, CBSD-L: cassava brown streak disease on leaves, CBSD-S: cassava brown streak disease on stem, CBSD-RN: cassava brown streak disease root necrosis, FSRY: fresh storage root yield, TB: total biomass, HI: harvest index, DMC: dry matter content, β-C: β-Carotene, PPD: postharvest physiological deterioration.

### 3.4 Specific combining ability for β-Carotene, delayed postharvest physiological deterioration and farmers’ preferred traits of cassava

The SCA effects of F1 families were estimated for specific traits. The family Garukunsubire x Gahene had desirable significant (p < 0.05) negative SCA for CMD, and no CMD symptoms. The families Mavoka x Mushedile and Gahene x Ndamirabana recorded the least CBSD-L (1.7) with significant (p < 0.05) negative SCA. The family Garukunsubire x Gahene had positive SCA for FSRY, and the highest average FSRY (13.7 t ha^−1^), while the family Mavoka x Mushedile had the lowest average FSRY (3.9 t ha^−1^), with significant (p < 0.05) negative SCA (-4.40). The family Garukunsubire x Gahene had the highest average of TB (3.63kgper plant) and significant (p < 0.05) positive SCA, while the family Garukunsubire x Ndamirabana showed the highest HI (0.34), with significant positive SCA. The family Mavoka x Garukunsubire had the highest average of DMC (35.9%), with highly significant (p < 0.001) positive SCA, while the family Mavoka x Ndamirabana recorded the lowest average of DMC (28.4%), with significant (p < 0.001) negative SCA (-3.62). The family of Mavoka x Garukunsubire had significant (p < 0.001) positive SCA for β-Carotene, and the highest average β-C. The family Mavoka x Ndamirabanahad the least PPD (13.3%) recorded after one week of storage (Table 5).

**Table 5:** Means and specific combining ability effects of 15 families of cassava clones for important traits generated from 6 × 6 half-diallel cross.

Families	CMD-S		CBSD-L		CBSD-S		CBSD-RN		FSRY (t ha^−1^)	
	$\bar{\text{X}}$	SCA	$\bar{\text{X}}$	SCA	$\bar{\text{X}}$	SCA	$\bar{\text{X}}$	SCA	$\bar{\text{X}}$	SCA
G1xG2	1.39	-0.01	1.98	-0.07	2.16	0	2.18	0.05	7.28	-3.55
G1xG3	1.46	0.05	2.18	0.14	2.14	-0.1	2.3	0.08	13.13	3.87
G1xG4	1.84	0.24*	1.73	-0.22*	2.12	0.02	2.13	-0.16	3.9	-4.40*
G1xG6	1.31	-0.02	2.08	0.01	2.28	0.02	2.4	0.02	11.19	1.27
G1xG7	1	-0.26*	2.27	0.13	2.44	0.05	2.3	0	13.66	2.8
G2xG3	1.26	-0.27*	2.12	0.13	2.27	0.16	2.01	0.12	6.36	-2.2
G2xG4	1.94	0.21	1.79	-0.1	1.86	-0.09	1.98	0.02	11.7	4.08*
G2xG6	1.44	-0.02	2	-0.01	2.13	0.01	1.82	-0.23	10.36	1.14
G2xG7	1.5	0.1	2.15	0.06	2.16	-0.08	2	0.03	10.68	0.52
G3xG4	1.56	-0.16	1.96	0.08	2.18	0.14	2.02	-0.02	5.94	-0.09
G3xG6	1.87	0.41**	1.77	-0.22*	1.9	-0.29*	1.99	-0.14	8.16	0.52
G3xG7	1.36	-0.02	1.9	-0.15	2.41	0.08	2.02	-0.03	6.48	-2.09
G4xG6	1.22	0.42***	2.17	-0.26**	2.16	-0.11	2.47	-0.25	6.03	0.65
G4xG7	1.72	0.13	1.94	-0.02	1.98	-0.19	2.05	-0.08	8.67	1.05
G6xG7	1.36	0.05	2.06	-0.03	2.46	0.13	2.31	0.08	6.93	-2.29
*Means*	*1.48*		*2.01*		*2.18*		*2.13*		*8.7*	*0.09*
*SE*	*0.046*		*0.032*		*0.048*		*0.092*		*0.666*	
*P Value*	<*.001*		<*.001*		<*.001*		<*.001*		<*.001*	
Families	TB (kg plant^−1^)		HI		DMC (%)		β-C (mg 100g^−1^)		PPD (%)	
	$\bar{\text{X}}$	SCA	$\bar{\text{X}}$	SCA	$\bar{\text{X}}$	SCA	$\bar{\text{X}}$	SCA	$\bar{\text{X}}$	SCA
G1xG2	2.39	-0.57	0.29	-0.03	35.9	3.42***	1.47	5.49***	32.5	17.68***
G1xG3	3.2	0.63*	0.34	0.02	33.28	0.35	0.26	-3.97***	24	-3.36
G1xG4	1.62	-1.02*	0.23	-0.02	34.65	1.28	0.63	-0.36	35.83	3.72
G1xG6	3.04	0.23	0.33	0.02	28.36	-3.62***	0.46	-2.94**	13.33	-10.06***
G1xG7	3.63	0.72*	0.33	0	30.75	-1.42	0.79	1.78	16.66	-7.98**
G2xG3	2.25	-0.31	0.27	-0.04	34.04	-0.84	0.20	-1.48	24.16	-7.61**
G2xG4	3.29	0.63*	0.34	0.06*	33.26	-2.06*	0.32	-1.54	34.16	-2.35
G2xG6	2.91	0.1	0.33	0.01	33.36	-0.58	0.50	1.02	25	-2.81
G2xG7	3.05	0.14	0.32	-0.01	34.21	0.07	0.19	-3.49***	24.16	-4.9
G3xG4	2.41	0.16	0.23	-0.01	35.87	0.09	0.00	1.85	50	0.92
G3xG6	2.45	0.04	0.32	0.02	35.5	1.1	0.00	1.85	50.17	9.81***
G3xG7	1.98	-0.52	0.32	0	33.86	-0.71	0.00	1.74	41.85	0.23
G4xG6	2.57	-0.08	0.21	0.04	35.7	-0.86	0.00	-0.08	39.16	5.94*
G4xG7	2.73	0.14	0.29	0.02	34.85	-0.17	0.00	-0.03	50	3.64
G6xG7	2.26	-0.47	0.29	-0.02	35.88	2.24*	0.00	-0.01	46.66	9.01**
*Means*	*2.65*		*0.29*		*33.96*		*0.32*		*33.84*	*0.79*
*SE*	*0.108*		*0.01*		*0.35*		*0.049*		*1.514*	
*P Value*	<*.001*		<*.001*		<*.001*		<*.001*		<*.001*	

SE: standard error, $\bar{\text{X}}$:means,G1: Mavoka, G2: Garukunsubire, G3: Gahene, G4: Mushedile, G6: Ndamirabana, G7: Gitamisi, SCA: specific combining ability, CMD-S: cassava mosaic disease severity, CBSD-L: cassava brown streak disease on leaves, CBSD-S: cassava brown streak disease on stem, CBSD-RN: cassava brown streak disease root necrosis, FSRY: fresh storage root yield, TB: total biomass, HI: harvest index, DMC: dry matter content, β-C: β-Carotene, PPD: postharvest physiological deterioration.

### 3.5 Estimates of mid-parent heterosis for selected traits of cassava clones across two locations

The family Mavoka x Garukunsubire expressed the highest positive heterosis for CMD, DMC and β-C. Out of 15 families, only three families (Movaka x Mushedile, Mushedile x Ndamirabana and Mushedile x Gitamisi) had desirable positive mid-parent heterosis for CBSD-S and CBSD-RN resistance. This indicates that Mushedile could be the better parent for improving cassava resistance to CBSD. In terms of FRSY, the families Mavoka x Gahene, Garukunsubire x Gahene and Gahene x Gitamisi had the highest positive mid-parent heterosis, while the remaining families expressed a negative heterosis for FRSY (Table 6). The heterosis for β-C was highly positive for all families with parent Garukunsubire in common, indicating that it could be a good combiner to improve β-C. The mid-parent heterosis for PPD was positive for the families Garukunsubire x Gitamisi, Mavoka x Mushedile and Ndamiraba x Gitamisi, while most of the families expressed the desirable negative heterosis (Table 6).

**Table 6:** Mid-parents heterosis for important cassava traits evaluated at clonal stage across two sites.

Families	CMD		CBSD-S		CBSD-RN		FSRY (t ha^−1^)	
	Mean	MPH	Mean	MPH	Mean	MPH	Mean	MPH
G1xG2	1.39	39.33	2.16	-38	2.18	-41.65	7.28	-51.12
G1xG3	1.46	-46.89	2.14	-38.62	2.30	-34.22	13.13	33.71
G1xG4	1.84	-17.83	2.12	6.29	2.13	6.62	3.90	-82.05
G1xG6	1.31	4.89	2.28	-23.92	2.40	-3.61	11.19	-38.73
G1xG7	1.00	-50	2.44	-2.05	2.30	-23.27	13.66	3.56
G2xG3	1.26	-54.06	2.27	-43.05	2.01	-52.63	6.36	-14.89
G2xG4	1.94	-13.36	1.86	-25.36	1.98	-27.68	11.70	-39.75
G2xG6	1.44	15.56	2.13	-39.12	1.82	-43.88	10.36	-34.9
G2xG7	1.50	-25	2.16	-27.86	2.00	-33.02	10.68	-1.54
G3xG4	1.56	-60.89	2.18	-12.68	2.02	-19.01	5.94	-58.53
G3xG6	1.87	-37.39	1.90	-45.67	1.99	-33.45	8.16	-24.67
G3xG7	1.36	-63.56	2.41	-19.58	2.02	-42.03	6.48	12.43
G4xG6	1.22	-45.37	2.16	8.02	2.47	65.17	6.03	-73.52
G4xG7	1.72	-47.06	1.98	32.49	2.05	2.68	8.67	-50.99
G6xG7	1.36	-39.27	2.46	-1.43	2.31	-7.51	6.93	-51.18
G1	1		3		3		17.3	
G2	1		4		4.5		12.6	
G3	4.5		4		4		2.4	
G4	3.5		1		1		26.3	
G5	1.5		3		2		19.3	
G6	3		2		3		9.13	
Families	HI		DMC (%)		β-C (mg 100 g^−1^)		PPD (%)	
	Mean	MPH	Mean	MPH	Mean	MPH	Mean	MPH
G1xG2	0.29	8.14	35.90	29.64	1.47	117.8	32.50	0
G1xG3	0.34	18.22	33.28	12.72	0.26	-60.6	24.00	-20
G1xG4	0.23	-38.96	34.65	18.54	0.63	-4.6	35.83	10.26
G1xG6	0.33	-1.79	28.36	-0.86	0.46	-30.4	13.33	-46.67
G1xG7	0.33	31.53	30.75	3.46	0.79	19.6	16.66	-44.44
G2xG3	0.27	11.73	34.04	8.94	0.20	1190.3	24.16	-53.97
G2xG4	0.34	-1.97	33.26	7.44	0.32	1964.5	34.16	-37.88
G2xG6	0.33	11.52	33.36	10	0.50	3125.8	25.00	-47.37
G2xG7	0.32	49.76	34.21	8.79	0.19	1125.8	24.16	-53.97
G3xG4	0.23	-34.86	35.87	9.4	0.00	0.0	50.00	-4.76
G3xG6	0.32	3.36	35.50	10.38	0.00	0.0	50.17	11.5
G3xG7	0.32	38.85	33.86	1.78	0.00	0.0	41.85	-16.3
G4xG6	0.21	-48.53	35.70	12.02	0.00	0.0	39.16	-17.54
G4xG7	0.29	-11.91	34.85	5.67	0.00	0.0	50.00	-4.76
G6xG7	0.29	3.49	35.88	10.91	0.00	0.0	46.66	3.7
G1	0.31		26		1.32		10	
G2	0.23		29.4		0.03		55	
G3	0.27		33.1		0		50	
G4	0.47		32.5		0		55	
G6	0.37		31.3		0		40	
G7	0.2		33.5		0		50	

MPH: mid-parent heterosis, G1: Mavoka, G2: Garukunsubire, G3: Gahene, G4: Mushedile, G6: Ndamirabana, G7: Gitamisi, SCA: specific combining ability, CMD-S: cassava mosaic disease severity, CBSD-S: cassava brown streak disease on stem, CBSD-RN: cassava brown streak disease root necrosis, FSRY: fresh storage root yield,HI: harvest index, DMC: dry matter content, β-C: β-Carotene, PPD: postharvest physiological deterioration.

### 3.6 Selection of best clones

**Fig. 1:**
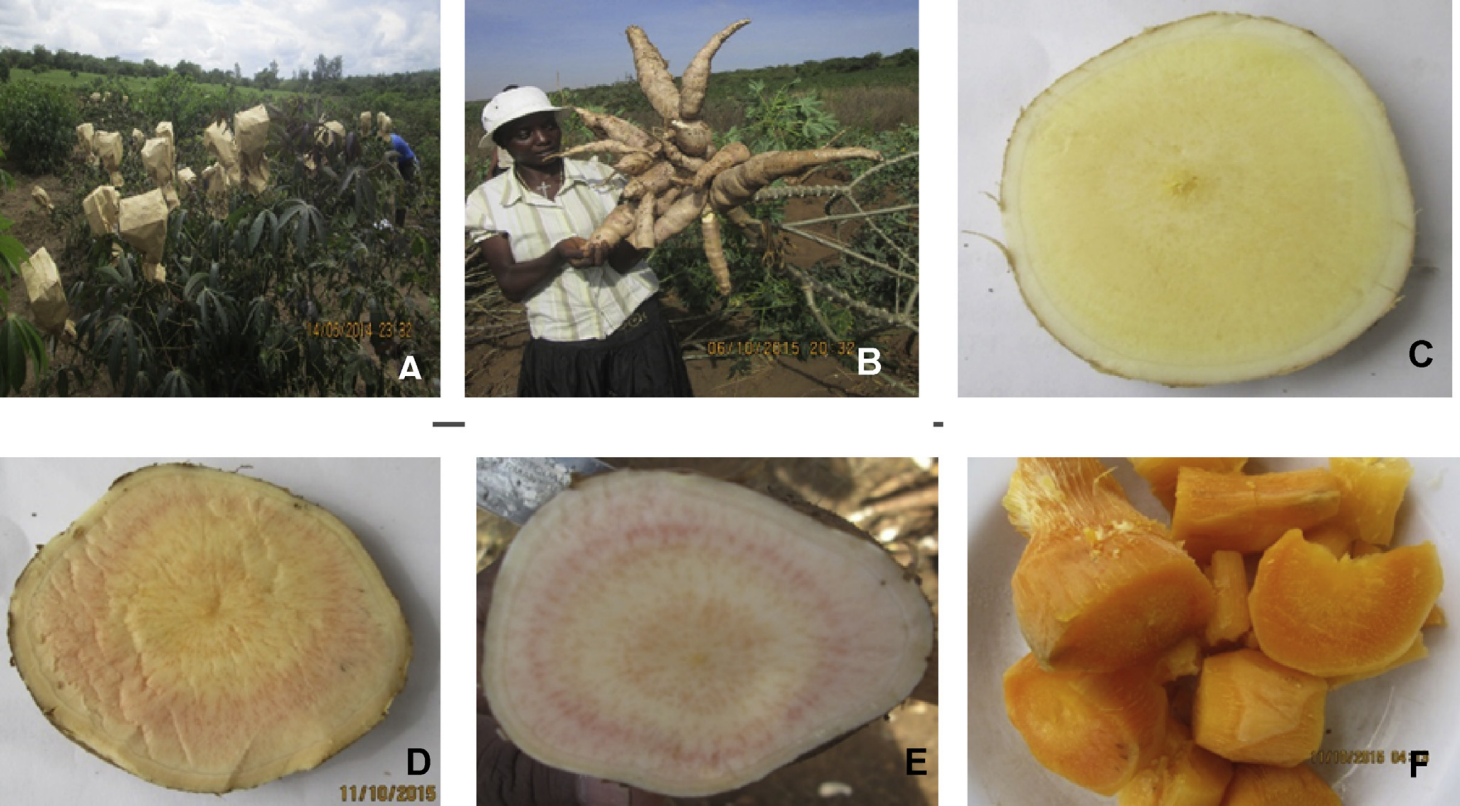
Developed cassava clones with high carotenoids and delayed PPD. A: Flowers covered to avoid free cross pollination, B: new clone with high yield, C: Deep yellow of cassava pulp, D and E: Orange and or pink of cassava pulp, F: Orange fleshed cooked cassava.

Top 15 clones were selected using a selection index based on the key four traits (FRSY, CBSD-RN, β-carotene and PPD). Most of the selected clones had in common parent Mavoka probably due to its high carotenoid content inducing flesh colour ranging from white to orange (Fig. 1).

Table 7 shows the ranks of 15 best clones for advancement for yield trials at multi-locations.

**Table 7:** Top 15 clones selected based on the key four traits.

Ranks	Clones	Pedigree	FSRY	CBSD-RN	β-C	PPD	SI
			$\bar{\text{X}}$	$\bar{\text{X}}$	$\bar{\text{X}}$	$\bar{\text{X}}$	
1	426	G1xG6	41.7	4.0	1.65	8	185.6
2	96	G1xG6	40.5	3.0	1.65	8	183.6
3	670	G1xG2	31.4	2.0	6.12	5	161.5
4	115	G1xG3	44.0	1.0	0.03	30	160.3
5	401	G1xG5	30.3	2.0	1.32	10	131.6
6	183	G2xG6	45.6	3.0	0.00	60	100.2
7	216	G1xG6	23.6	2.0	1.32	10	98.1
8	93	G1xG6	23.8	1.0	0.15	15	89.6
10	272	G1xG6	25.5	1.0	0.15	20	88.1
11	78	G1xG3	19.0	1.0	1.32	10	79.1
12	52	G1xG3	17.9	1.0	1.65	8	78.6
13	52	G1xG3	19.5	2.0	1.32	10	77.6
14	79	G1xG5	26.8	4.0	0.03	30	62.3
15	79	G1xG5	26.8	4.0	0.03	30	62.3

$\bar{\text{X}}$: means, CBSD-RN: cassava brown streak disease root necrosis, FSRY: fresh storage root yield,β-C: β-Carotene, PPD: postharvest physiological deterioration, SI: Selection index.

## 4 Discussion

This study was conducted on 450 clones of 15 families generated from 6 × 6 half-diallel mating design. The F_1_ clones exhibited considerable phenotypic variability among families and progenies for the evaluated traits, such as FSRY, β-C, DMC, TBM, CMD-S, HI, CBSD-S, CBSD-RN and PPD. Some F_1_ progenies had higher amounts of β-C and higher PPD tolerance than their parents, which could be attributed to the transgressive segregation and heterosis, which are desirable for the improvement of most cassava traits. Similar findings reported by Tumuhimbise (2013) and Njenga et al. (2014) indicated that some cassava progenies outperformed their parents in terms of various traits, including FRSY and pulp/flesh colour (an indication of β-C content).

The environments did not exhibit a significant influence on the expression of β-C and PPD, indicating the expression of such traits are mostly genetically controlledby genetic background of the plant. Tumuhimbise et al. (2015) reported a low environmental effect on PPD expression, while the low environmental effects on β-C agrees with the findings of many authors (Akinwale et al., 2011; Rodriguez-Amaya, 2010; Ssemakula and Dixon, 2007), who indicated that the accumulation of β-C is predominately governed by genetic effect, with a low GxE interaction. The families’ mean squares exhibited a significant difference for all traits, indicating significant variation among families. The environment × family interaction effects were significant for most traits, except HI and PPD, indicating that selection for these two traits cannot be performed solely at one location. The remaining traits were stable and could be selected at either location. The significant G × E interaction effects indicated that most traits hadunstable performance across two locations. These findings agree with many authors (Baiyeri et al., 2008; Njoku et al., 2015; Ntawuruhunga and Dixon, 2010; Tumuhimbise et al., 2015; Were et al., 2012), who reported significant G × E interaction effects for most agronomic and morphological cassava traits. Only two sites were used because of the small number of stakes available and thus, further studies on G × E interaction are needed.

The GCA and SCA for both β-C and PPD were highly significant, indicating the role of additive and non-additive gene action in controlling such traits. The relative importance of additive and non-additive gene effects revealed that the pulp traits (CBSD-RN, β-C and PPD) were highly influenced (over 50% of variability) by GCA effects, indicating that such traits are predominantly controlled by additive gene action. A similar finding was reported by Tumuhimbise (2013) and Kulembeka et al. (2012), who indicated that CBSD-RN severity and PPD are predominantly controlled by additive gene action. The β-C was controlled by additive gene action, which is desirable as the trait that can be improved through recurrent selection. This was supported by Njenga et al. (2014) who reported that the pulp colour of the cassava storage root is positively controlled by additive gene action. Ceballos et al. (2013) and Nduwumuremyi et al., 2016a, Nduwumuremyi et al., 2016b reported that carotene can be selected through recurrent selection in the cassava breeding scheme. The GCA results indicated that the pulp traits are highly heritable and should react positively to selection. This agrees with Parkes et al. (2013), who reported that the traits with a predominance of additive gene action are highly heritable and react positively to selection.

The viral diseases and yield traits (CMD, CBSD-L, CBSD-S, TB, FSRY, HI and DMC) were considerably influenced (over 50% of variability) by SCA effects, which showed a predominance of non-additive gene action in controlling these traits. Several authors (Chipeta et al., 2015; Kulembeka et al., 2012; Tumuhimbise, 2013; Were et al., 2012) reported on the non-additive gene action for FRSY and most of the cassava traits. The non-additive gene action found for CMD disagreed with Tumuhimbise (2013) and Parkes et al. (2013) also reported that CMD resistance is predominantly controlled by additive gene action.

The GCA for parents indicated that genotype Mavoka had a significant desirable positive GCA for β-C and FRSY, undesirable significant negative GCA for DMC, and a desirable significant negative GCA for PPD. The genotype Garukunsubire presented similar attributes, indicating the ability of the both parents to improve the level of β-Carotene and delayed PPD, when combined in a hybridization scheme. The improvement of β-C content in the cassava population, using Mavoka as progenitor, could be used to concurrently improve yield and delayed PPD, but could lead to a reduction in the dry matter content. The findings on the negative correlation between carotenoid and dry matter was reported by many authors (Akinwale et al., 2010; Ceballos et al., 2012; Esuma et al., 2012; Nduwumuremyi et al., 2016a, Nduwumuremyi et al., 2016b; Vimala et al., 2009), which could negatively affect the farmers’ adoption. The antioxidant propreties of carotenoid could delay the PPD by protecting the wounded part of the storage root against reactive oxygen, as reported by many authors (Azqueta and Collins, 2012; Edge et al., 1997; Giuliano, 2014; Nduwumuremyi et al., 2016a, Nduwumuremyi et al., 2016b; Priya and Siva, 2014; Xu et al., 2013; Zidenga et al., 2012), and could promote the adoption of improved carotenoid cassava clones.

In terms of CMD, the clones from the families Garukunsubire x Gahene and Garukunsubire x Mushedile had the desirable high negative SCA, indicating that Garukunsubire could be a good combiner for CMD resistance. The individuals from the family Mushedile x Ndamirabaha, followed by Mavoka x Mushedile and Garukunsubire x Gitamisi, had a desirable high negative SCA (-0.26, -0.22, respectively) for CBSD-L. The family Mavoka x Garukunsubire had the highest average DMC (35.9%), and a desirable significant positive SCA, while the family Mavoka x Ndamirabana recorded the lowest average of DMC (28.3%), and an undesirable significant negative SCA (-3.62). The family Mavoka x Garukunsubirehad the highest average β-Carotene, with a desirable positive SCA, while the family Mavoka x Ndamirabana had the lowest PPD (13.3%) after one week of storage and a desirable negative SCA (-10.06).

The progenies from family Mavoka x Garukunsubire expressed the highest positive heterosis for CMD, DMC and β-C. The high positive heterosis for DMC in this family is an interesting scenario, which could be linked to transgressive segregation, because one of the parents was a bad combiner for DMC. The progenies from three families (Movaka x Mushedile, Mushedile x Ndamirabana and Mushedile x Gitamisi) had a desirable positive mid-parent heterosis for CBSD-S and CBSD-RN resistance, indicating that Mushedile could be used for improving cassava resistance to CBSD. In terms of FRSY, the families Mavoka x Gahene, Garukunsubire x Gahene and Gahene x Gitamisi had the highest positive mid-parent heterosis, indicating that Gahene could be a good combiner for FRSY. The mid-parent heterosis for PPD was positive for the families Garukunsubire x Gitamisi, Mavoka x Mushedile and Ndamiraba x Gitamisi, while most of the families expressed negative heterosis. The heterosis for FRSY, DMC, CMD, CBSD, β-C and PPD indicates the genetic diversity of the parents used.

## 5 Conclusion

This study gave an insights into the feasibility of improvement of most important cassava traits, and provides the foundation for a cassava breeding program. It was noted that the transgressive segregation and heterosis for β-C and DMC are important aspect for cassava postharvest and nutritional improvement. The β-C and PPD expression were predominated by the additive gene action, while the CMD and CBSD were influenced by the non-additive gene action. The most of the clones were seriously affected by CBSD. Therefore, more investigation is needed to identify new sources of resistance to CBSD and CMD, and to develop a protocol for rapid multiplication of cuttings, to facilitate dissemination of newly-developed cassava hybrids.

### 5.1 Key message

The revealed transgressive segregation and heterosis for β-C and DMC is a desirable aspect for cassava postharvest and nutritional improvement. The additive gene action plays a great role for expression of β-C and PPD, while the non-additive gene action influenced the expression of CMD and CBSD.

## Declarations

### Author contribution statement

Nduwumuremyi Athanase: Conceived and designed the experiments; Performed the experiments; Analyzed and interpreted the data; Wrote the paper.

Melis Rob: Analyzed and interpreted the data; Wrote the paper.

### Funding statement

This work was supported by the Alliance for a Green Revolution in Africa (AGRA) through the African Centre for Crop Improvement (ACCI).

### Competing interest statement

The authors declare no conflict of interest.

### Additional information

No additional information is available for this paper.

## References

[bib1] Akinwale M., Aladesanwa R., Akinyele B., Dixon A., Odiyi A. (2010). Inheritance of ß-carotene in cassava (*Manihot esculenta* crantz). Int. J. Genet. Mol. Biol..

[bib2] Akinwale M., Akinyele B., Odiyi A., Dixon A. (2011). Genotype x Environmnet interaction and yield performance of 43 improved cassava (*Manihot esculenta* Crantz) genotypes at three Agro-Climatic zones in Nigeria. Biotechnol. J..

[bib3] Azqueta A., Collins A.R. (2012). Carotenoids and DNA damage. Mutat. Res. Fund Mol. Mech. Mutagen.

[bib4] Baiyeri K., Edibo G., Obi I., Ogbe F., Egesi C., Eke-Okoro O., Okogbenin E., Dixon A. (2008). Growth, yield and disease responses of 12 cassava genotypes evaluated for two cropping seasons in a derived savannah zone of South-Eastern Nigeria. Agro-Science. J. Trop. Agric. Food Environ. Ext..

[bib5] Burgos B., Carpio R., Sanchez C., Paola S., Eduardo P., Espinoza J., Grüneberg W. (2009). A Colour Chart to Screen for High β-carotene in OFSP Breeding. The 15th Triennial Symposium of the International Society for Tropical Root Crops.

[bib6] Ceballos H., Iglesias C.A., Perez J.C., Dixon A.G. (2004). Cassava breeding: opportunities and challenges. Plant Mol. Biol..

[bib7] Ceballos H., Luna J., Escobar A.F., Ortiz D., Pérez J.C., Sánchez T., Pachón H., Dufour D. (2012). Spatial distribution of dry matter in yellow-fleshed cassava roots and its influence on carotenoid retention upon boiling. Food Res. Int..

[bib8] Ceballos H., Morante N., Sánchez T., Ortiz D., Aragón I., Chávez A., Pizarro M., Calle F., Dufour D. (2013). Rapid cycling recurrent selection for increased carotenoids content in cassava roots. Crop Sci..

[bib9] Chávez A.L., Sánchez T., Jaramillo G., Bedoya J.M., Echeverry J., Bolaños E.A., Ceballos H., Iglesias C.A. (2005). Variation of quality traits in cassava roots evaluated in landraces and improved clones. Euphytica.

[bib10] Chipeta M.M., Bokosi J.M., Saka V.W. (2015). Genetic analysis in a six parent diallel cross of cassava (*Manihot esculenta* Crantz). Afr. J. Agric. Res..

[bib11] Edge R., Mcgarvey D., Truscott T. (1997). The carotenoids as anti-oxidants—a review. J. Photochem. Photobiol. B Biol..

[bib12] Ephraim N., Yona B., Evans A., Sharon A., Titus A. (2015). Effect of cassava brown streak disease (CBSD) on cassava (*Manihot esculenta* Crantz) root storage components, starch quantities and starch quality properties. Int. J. Plant Physiol. Biochem..

[bib13] Esuma W., Rubaihayo P., Pariyo A., Kawuki R., Wanjala B., Nzuki I., Harvey J.J., Baguma Y. (2012). Genetic diversity of pro-Vitamin A Cassava in Uganda. J. Plant Stud..

[bib14] Fukuda W., Guevara C., Kawuki R., Ferguson M. (2010). Selected Morphological and Agronomic Descriptors for the Characterization of Cassava.

[bib15] Giuliano G. (2014). Plant carotenoids: genomics meets multi-gene engineering. Curr. Opin. Plant Biol..

[bib16] Glover M.A., Willmot D.B., Darrah L.L., Hibbard B.E., Zhu X. (2005). Diallel analyses of agronomic traits using Chinese and US maize germplasm. Crop Sci..

[bib17] Griffing B. (1956). Concept of general and specific combining ability in relation to diallel crossing systems. Aust. J. Biol. Sci..

[bib18] Hillocks R., Raya M., Thresh J. (1996). The association between root necrosis and above-ground symptoms of brown streak virus infection of cassava in southern Tanzania. Int. J. Pest Manag..

[bib19] IITA (2016). Cassava Crop.

[bib19a] Kawano K., Fehr W.R., Hadley H.H. (1980). Cassava. Hybridisation of Crop Plants.

[bib20] Kawano K., Fukuda W.M.G., Cenpukdee U. (1987). Genetic and environmental effects on dry matter content of cassava root. Crop Sci..

[bib21] Kiaya V. (2014). Post-harvest Losses and Strategies to Reduce Them.

[bib22] Kulembeka H., Ferguson M., Herselman L., Kanju E., Mkamilo G., Masumba E., Fregene M., Labuschagne M. (2012). Diallel analysis of field resistance to brown streak disease in cassava (*Manihot esculenta* Crantz) landraces from Tanzania. Euphytica.

[bib23] Mendes U.C., Oliveira A.S., Reis E.F.D. (2015). Heterosis and combining ability in crosses between two groups of open-pollinated maize populations. Crop Breed. Appl. Biotechnol..

[bib24] Morante N., Sánchez T., Ceballos H., Calle F., Pérez J.C., Egesi C., Cuambe C.E., Escobar A.F., Ortiz D., Chávez A.L., Fregene M. (2010). Tolerance to postharvest physiological deterioration in cassava roots. Crop Sci..

[bib25] Nduwumuremyi A., Melis R., Shanahan P., Asiimwe T. (2016). Introgression of antioxidant activity into cassava (*Manihot esculenta* C.): an effective technique for extending fresh storage roots shelf-life. Plant Breed..

[bib26] Nduwumuremyi A., Melis R., Shanahan P., Asiimwe T. (2016). Participatory appraisal of preferred traits, production constraints and postharvest challenges for cassava farmers in Rwanda. Food Secur..

[bib27] Nduwumuremyi A., Tongoona P., Habimana S. (2013). Mating designs: helpful tool for quantitative plant breeding analysis. J. Plant Breed. Genet..

[bib28] Njenga P., Edema R., Kamau J. (2014). Combining ability for beta-carotene and important quantitative traits in a cassava F1 population. J. Plant Breed Crop Sci..

[bib29] Njoku D.N., Gracen V.E., Offei S.K., Asante I.K., Egesi C.N., Kulakow P., Ceballos H. (2015). Parent-offspring regression analysis for total carotenoids and some agronomic traits in cassava. Euphytica.

[bib30] Ntawuruhunga P., Dixon A.G. (2010). Quantitative variation and inter-relationship between factors influencing cassava yield. J. Appl. Biosci..

[bib31] Parkes E.Y., Fregene M., Dixon A., Boakye-Peprah B., Labuschagne M.T. (2013). Combining ability of cassava genotypes for cassava mosaic disease and cassava bacterial blight, yield and its related components in two ecological zones in Ghana. Euphytica.

[bib32] Priya R., Siva R. (2014). Phylogenetic analysis and evolutionary studies of plant carotenoid cleavage dioxygenase gene. Gene.

[bib33] Rodriguez-Amaya D.B. (2010). Quantitative analysis in vitro assessment of bioavailability and antioxidant activity of food carotenoids-A review. J. Food Compos. Anal..

[bib34] Rwegasira G.M., Rey C.M. (2012). Response of selected cassava varieties to the incidence and severity of cassava brown streak disease in Tanzania. J. Agric. Sci..

[bib35] Ssemakula G., Dixon A. (2007). Genotype x environment interaction, stability and agronomic performance of carotenoid-rich cassava clones. Sci. Res. Essays.

[bib36] Tumuhimbise R. (2013). Breeding and Evaluation of Cassava for High Storage Root Yield and Early Bulking in Uganda.

[bib37] Tumuhimbise R., Melis R., Shanahan P. (2014). Diallel analysis of early storage root yield and disease resistance traits in cassava (*Manihot esculenta* Crantz). Field Crop. Res..

[bib38] Tumuhimbise R., Melis R., Shanahan P. (2015). Genetic variation in cassava for postharvest physiological deterioration. Arch. Agron Soil Sci..

[bib39] Upadhyay M., Jaiswal H. (2015). Combining ability analysis for yield and earliness in hybrid rice (*Oryza sativa* L.). Asian J. Crop Sci..

[bib40] Vimala B., Nambisan B., Thushara R., Unnikrishnan M. (2009). Variability of carotenoids in yellow-fleshed cassava (*Manihot esculenta* Crantz) clones. Gene Conserve.

[bib41] Were W., Shanahan P., Melis R., Omari O. (2012). Gene action controlling farmer preferred traits in cassava varieties adapted to mid-altitude tropical climatic conditions of western Kenya. Field Crop. Res..

[bib42] Wheatley C., Lozano C., Gomez G., Cock J.H., Reyes J.A. (1985). Post-harvest deterioration of cassava roots. Cassava: Research, Production and Utilization.

[bib43] Xu J., Duan X., Yang J., Beeching J.R., Zhang P. (2013). Enhanced reactive oxygen species scavenging by overproduction of superoxide dismutase and catalase delays postharvest physiological deterioration of Cassava storage roots. Plant Physiol..

[bib44] Zhang Y., Kang M.S., Lamkey K.R. (2005). Diallel-SAS05. Agron. J..

[bib45] Zhao X., Li B., Zhang K., Hu K., Yi B., Wen J., Ma C., Shen J., Fu T., Tu J. (2016). Breeding signature of combining ability improvement revealed by a genomic variation map from recurrent selection population in *Brassica napus*. Sci. Rep..

[bib46] Zidenga T., Leyva-Guerrero E., Moon H., Siritunga D., Sayre R. (2012). Extending cassava root shelf-life via reduction of reactive oxygen species production. Plant Physiol..

